# Pre-hospital management of severe traumatic brain injury- compliance with guidelines

**DOI:** 10.1186/1757-7241-22-S1-P1

**Published:** 2014-07-07

**Authors:** Rebecka M Rubenson Wahlin, Johannes Gustavsson, Maaret K Castrén

**Affiliations:** 1 Department of Clinical Science and Education, Södersjukhuset, Karolinska Institute, Stockholm, Sweden

## Background

Traumatic brain injury (TBI) is a major cause of death and disability. Guidelines for pre-hospital management of severe TBI have been published but research has shown that pre-hospital management often differs from that recommended in guidelines.

## Aim

To assess compliance with guidelines in the pre-hospital management of severe TBI in Stockholm, in assessment and/or treatment of airway, ventilation, oxygenation, blood pressure, fluid resuscitation, GCS, and pupil properties.

## Method

The study was a cross sectional observational study based on data from trauma registry and ambulance records. Included patients (n=101) were adult (age > 15 years), with Injury Severity Score > 9 and GCS 3-8 (severe TBI caused by blunt trauma), transported by ambulance or helicopter and admitted to the trauma center at Karolinska University Hospital in Stockholm between January 1^st^ and December 31^st^, 2008. TBI were confirmed by computer tomography and with a corresponding ICD-10 diagnosis (s06.0-06.91). The pre-hospital management considered compliant with guidelines only if the assessment or the treatment was documented in the ambulance journal. A 65% criterion was used as a lower limit for acceptable compliance.

## Results

Compliance with guidelines was; oxygenation assessment 90.1%, hypoxia treatment 100.0 %, blood pressure assessment 88.1%, hypotension treatment 90.0%, Glasgow Coma Scale 76.2% and pupil properties assessment 93.1%.

## Conclusion

The pre-hospital management of severe TBI was to a large extent compliant with the guidelines in Stockholm and compliance was higher compared with that of previous studies.
